# Bacterial Diversity Based on a 16S rRNA Gene Amplicon Data Set from a High-Altitude Crater Lake and Glacial Samples of the Iztaccihuatl Volcanic Complex (Mexico)

**DOI:** 10.1128/MRA.01636-18

**Published:** 2019-03-21

**Authors:** Rosa P. Calvillo-Medina, Juan P. Reyes-Grajeda, Vicente D. Moreno-Andrade, Luis Barba-Escoto, Victor Bautista-de Lucio, George H. Jones, Juan Campos-Guillén

**Affiliations:** a Laboratorio de Microbiología Molecular, Facultad de Química, Universidad Autónoma de Querétaro, Santiago de Querétaro, Querétaro, Mexico; b Facultad de Ciencias Naturales, Universidad Autónoma de Querétaro, Santiago de Querétaro, Querétaro, Mexico; c Laboratorio de Estructura de Proteínas, Instituto Nacional de Medicina Genómica, Mexico City, Mexico; d Departamento de Microbiología y Proteómica, Instituto de Oftalmología Fundación Conde de Valenciana, Mexico City, Mexico; e International Maize and Wheat Improvement Center (CIMMYT), Sustainable Intensification Program, Texcoco de Mora, Estado de México, Mexico; f Department of Biology, Emory University, Atlanta, Georgia, USA; University of Maryland School of Medicine

## Abstract

Little is known about extremophilic microorganisms from glaciers found in subtropical regions, and to our knowledge, no reports have identified glacial bacteria in this ecosystem in Mexico. Herein, we report a 16S rRNA gene amplicon data set demonstrating bacterial diversity of three samples from the Iztaccihuatl volcanic complex (Mexico) with a total of 115,701 to 138,805 high-quality reads. The bacterial population was classified at the phylum level in all samples.

## Abstract (precis)

Little is known about extremophilic microorganisms from glaciers found in subtropical regions, and to our knowledge, no reports have identified glacial bacteria in this ecosystem in Mexico. Herein, we report a 16S rRNA gene amplicon data set demonstrating bacterial diversity of three samples from the Iztaccihuatl volcanic complex (Mexico) with a total of 115,701 to 138,805 high-quality reads.

## ANNOUNCEMENT

The Iztaccihuatl (“the white woman” in the ancient Nahuatl language) is a Pleistocene stratovolcano complex. It is an active volcano and hosts three glaciers and two crater lakes (1). Little is known about the microbially diverse populations in high-altitude tropical crater lakes and glaciers. What is apparent is that microorganisms can survive in these glacial conditions because they are highly adapted to extreme and stressful environments (2, 3). Analysis based on 16S rRNA gene amplicon data sets provides information about the bacterial ecology and biotechnological applications of these microorganisms (4).

Samples for 16S rRNA gene amplicon analysis were obtained in triplicate (22 June 2017) from the Monte de Venus crater lake at 4,950 meters above sea level (masl) (N19°09.810′, W098°38.277′; pH 6.6 and 2°C) and from the glaciers La Panza at 5,065 masl (N19°10.056′, W098°38.327′; pH 6.6 and −2°C) and El Pecho at 5,200 masl (N19°10.609′, W098°38.475′; pH 6.8 and −4°C).

Glacier samples were collected in sterile glass containers of 500 ml by boring holes to a depth of 60 cm using a sterile ice axe (sterilized on site using 96° ethanol and flaming). Crater lake samples (200 ml) underneath the frozen lake surface were obtained by cracking the ice surface to create a hole using a sterile ice axe and submerging sterile glass containers to a depth of 60 cm. In all samples, bottles were opened inside the holes or inside the lakes and filled with ice or with water. The containers were placed in a cooler and transferred to the laboratory the same day. Once in the lab, samples were stored overnight at 4°C (for melting of ice samples) and processed the next day. These samples were passed through 0.22-mm filters (Millipore) to recover bacterial cells (5). The filters were placed in lysis buffer (Zymo Research, Irvine, CA), and DNA extraction was performed with a soil and water microbe DNA extraction kit (ZymoBIOMICS DNA minikit; Zymo Research, Irvine, CA) following the kit’s instructions (6).

The V3 and V4 regions of the 16S rRNA genes were amplified with PCR using the 337FF and 805R primers (6). Amplicons of the 16S rRNA genes were sequenced. The purification, quantification, and sequencing of the amplicon library was done at Macrogen, Inc. (Seoul, Republic of Korea) and accomplished via the Illumina platform with a MiSeq system. The raw sequences were processed to obtain high-quality reads and to remove adaptor sequences, ambiguous reads, low-quality sequences, and reads shorter than 50 bp. The sequences then were uploaded to the service cloud for their analysis. The total numbers of high-quality reads were 115,701 from Monte de Venus, 117,207 from La Panza, and 138,805 from El Pecho. To identify operational taxonomic units (OTUs), 16S rRNA gene metagenomic libraries were clustered, and processed sequences were analyzed with the 16S biodiversity tool and classified with RDP tools version 2.12 (7), with 97% identity of bacterial diversity. Bioinformatics analysis was carried out with Geneious software v.9.1.8 (8).

**FIG 1:**
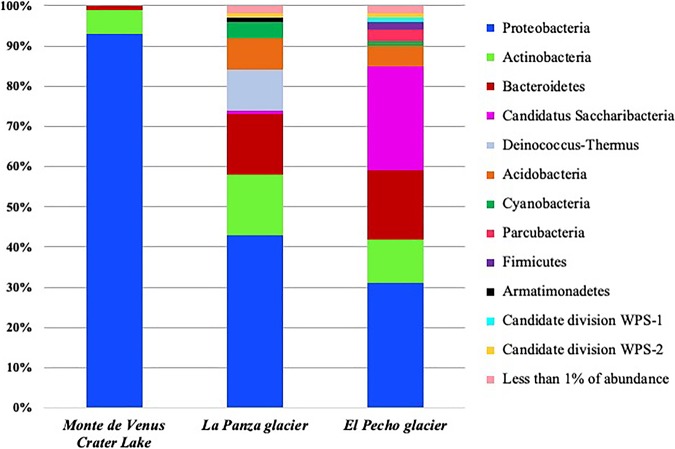
Bar chart of the bacterial diversity based on 16S rRNA gene amplicon analysis of samples from the Iztaccihuatl volcano. Each bar represents the phylum indicated by percent diversity from the three regions of the volcano that were sampled.

The resulting diagram (Fig. 1) shows that the most abundant OTUs in the taxonomic classification at the phylum level in the three samples were *Proteobacteria* (31 to 93%), followed by *Actinobacteria* (6 to 15%) and *Bacteroidetes* (1 to 17%). In Monte de Venus, members of *Proteobacteria* (93%), *Actinobacteria* (6%), and *Bacteroidetes* (1%) were found. In the La Panza glacier, members of *Proteobacteria* (43%), *Bacteroidetes* (15%), *Actinobacteria* (15%), *Deinococcus-Thermus* (10%), *Acidobacteria* (8%), *Cyanobacteria* (4%), *Armatimonadetes* (1%), candidate division WPS-2 (1%), and “*Candidatus* Saccharibacteria” (1%) were found. In the El Pecho glacier, *Proteobacteria* (31%), “*Candidatus* Saccharibacteria” (26%), *Bacteroidetes* (17%), *Actinobacteria* (11%), *Acidobacteria* (5%), *Parcubacteria* (3%), *Firmicutes* (2%), *Cyanobacteria* (1%), candidate division WPS-1 (1%), and candidate division WPS-2 (1%) were found.

The study represents, to our knowledge, the first report of the bacterial diversity based on a 16S rRNA gene amplicon data set from the Iztaccihuatl volcano samples, and it extends our knowledge of the diversity of extremophilic microorganisms from these tropical glaciers.

### Data availability.

The 16S rRNA gene amplicon data set was deposited in GenBank under the SRA accession numbers SRS3420169 (Monte de Venus crater lake), SRS3420167 (La Panza glacier), and SRS3420168 (El Pecho glacier).
